# Human IgG responses to *Aedes* mosquito salivary peptide Nterm-34kDa and its comparison to *Anopheles* salivary antigen (gSG6-P1) IgG responses measured among individuals living in Lower Moshi, Tanzania

**DOI:** 10.1371/journal.pone.0276437

**Published:** 2022-10-27

**Authors:** Nancy A. Kassam, Daniel Laswai, Neema Kulaya, Robert D. Kaaya, Debora C. Kajeguka, Christentze Schmiegelow, Christian W. Wang, Michael Alifrangis, Reginald A. Kavishe

**Affiliations:** 1 Kilimanjaro Christian Medical University College (KCMUCo), Moshi, Tanzania; 2 Muhimbili University of Health and Allied Sciences (MUHAS), Dar es Salaam, Tanzania; 3 Pan-African Malaria Vector Research Consortium, Moshi, Tanzania; 4 Centre for Medical Parasitology, Department of Immunology and Microbiology, University of Copenhagen and Department of Infectious Diseases, Copenhagen University Hospital (Rigshospitalet), Copenhagen, Denmark; Universiti Kebangsaan Malaysia, Faculty of Medicine, MALAYSIA

## Abstract

**Background:**

The level of human exposure to arbovirus vectors, the *Aedes* mosquitoes, is mainly assessed by entomological methods which are labour intensive, difficult to sustain at a large scale and are affected if transmission and exposure levels are low. Alternatively, serological biomarkers which detect levels of human exposure to mosquito bites may complement the existing epidemiologic tools as they seem cost-effective, simple, rapid, and sensitive. This study explored human IgG responses to an *Aedes* mosquito salivary gland peptide Nterm-34kDa in Lower Moshi, a highland area with evidence of circulating arboviruses and compared the *Aedes* IgG responses to *Anopheles* mosquitoes’ salivary antigen (GSG6-P1) IgG responses.

**Methods:**

Three cross-sectional surveys were conducted in 2019: during the first dry season in March, at the end of the rainy season in June and during the second dry season in September in five villages located in Lower Moshi. Blood samples were collected from enrolled participants above six months of age (age span: 7 months to 94 years) and analysed for the presence of anti-Nterm-34kDa IgG antibodies. Possible associations between Nterm-34kDa seroprevalence and participants’ characteristics were determined. Levels of IgG responses and seroprevalence were correlated and compared to the already measured IgG responses and seroprevalence of *Anopheles* mosquitoes’ salivary antigen, GSG6-P1.

**Results:**

During the first dry season, Nterm-34kDa seroprevalence was 34.1% and significantly increased at the end of the rainy season to 45.3% (Chi square (χ^2^) = 6.42 *p* = 0.011). During the second dry season, the seroprevalence significantly declined to 26.5% (χ^2^ = 15.12 *p*<0.001). During the rainy season, seroprevalence was significantly higher among residents of Oria village (adjusted odds ratio (AOR) = 2.86; 95% CI = 1.0–7.8; p = 0.041) compared to Newland. Moreover, during the rainy season, the risk of exposure was significantly lower among individuals aged between 16 and 30 years (AOR = 0.25; 95% CI = 0.1 = 0.9; p = 0.036) compared to individuals aged between 0 and 5 years. There was weak to moderate negative correlation between N-term 34kDa IgG and gSG6-P1 antigens. N-term 34kDa seroprevalence were higher compared to gSG6-P1 seroprevalence.

**Conclusion:**

The findings of this study support that IgG antibody responses towards the *Aedes* mosquito salivary peptide Nterm-34kDa are detectable among individuals living in lower Moshi and vary with season and geographical area. More individuals are exposed to *Aedes* mosquito bites than *Anopheles* mosquito and those exposed to *Aedes* bites are not necessarily exposed to *Anopheles* mosquitoes.

## Background

As of 2017 ~97 million cases of arboviral infections were estimated worldwide; with the main contributors being dengue virus (DENV) (96 mill.), chikungunya virus (CHIKV), zika virus, and yellow fever virus [1]. Specifically for dengue, the number of cases reported to the World Health Organisation (WHO) increased more than 10-fold over the last two decades; from 505,430 cases in 2000 to 5.2 million in 2019, while reported deaths increased from 960 to 4,030 between the year 2000 and 2015 [2]. There are however huge uncertainties to these numbers and in particular for sub-Saharan Africa (SSA), the data on dengue is patchy [3, 4]. A recent meta-analysis study reported the prevalence of DENV expressed either in terms of serologic markers of past and/or present infections (IgG or IgM) or confirmed acute infections (RNA) to be as high as 25%, 10% and 14%, respectively [5]. Over the last decade, Tanzania has encountered five dengue epidemics [6]. In addition to that, studies conducted in different parts of Tanzania have recorded serological prevalence of DENV, CHIKV and rift valley fever to vary between 1% and 38.2% [7–10], 3% and 46.8% [7, 9, 11–13] and 2.1% and 17.6% [12, 14–16], respectively. In particular, the business capital Dar es Salaam has been the epicentre for dengue outbreaks [17].

*Aedes* mosquitoes are the major vectors of arboviruses such as DENV and CHIKV, where *Aedes aegypti* and *Aedes albopictus* are the most important vectors [18]. The distribution of *Aedes* mosquitoes are mostly in the urban environment of the tropic and subtropics [19, 20], with *Aedes aegypti* being more common in rural settings and *Aedes albopictus* in urban settings [21]. As increased urbanisations are projected for the coming years, this will alter the availability of vector breeding habitats and thus, increase vector-human contact followed by an increased risk of arboviral disease transmission [22]. Moreover, climate change is expected to expand the transmission of arboviruses as warmer temperatures increases mosquito habitat suitability in regions that were previously too cold [22]. Optimal arbovirus transmission by *Ae*. *aegypti* peaks at 29°C [22]. In much of SSA, the temperature ranges between 25°C and 29°C, hence climate warming will increase suitable habitats for *Aedes* mosquitoes and increase the risk of arboviruses transmission including expansion into highland areas [22].

Arboviral vector transmission dynamics should be monitored closely to help predict the occurrence of epidemics, understand both individual and population transmission risk and constrain transmission by implementing effective vector control methods. Currently, the level of exposure to arbovirus vectors is mainly evaluated based on entomological methods such as identification of breeding sites, capture of adult mosquitoes by trapping and human landing catches [23]. Human landing catches assess risk by estimating direct levels of exposure to mosquito bites in human population [24] but puts the bait at risk for infections and thus, raises ethical concerns. Entomological methods are also labour intensive, difficult to sustain at a large scale and are affected when transmission and exposure levels are low, for instance during dry seasons, in high altitude areas and after vector control [24, 25].

Alternatively, serological biomarkers which detect levels of human exposure to mosquito bites may potentially complement or even replace existing epidemiologic tools as they seem cost-effective, simple, rapid, and sensitive [24]. Furthermore, serological biomarkers can estimate both individual and population levels of exposure and may be more appropriate for assessing the efficacy of vector control measures [24, 25].

During blood feeding, mosquitoes inject salivary peptides, some of which are functional and necessary for blood uptake. The peptide molecules are capable of initiating a specific human immunological response to produce targeting antibodies. For instance regarding malaria, several studies have found that immunological responses against *Anopheles* salivary biomarker, gSG6-P1 is a suitable proxy for exposure to *Anopheles* mosquito bites as IgG antibodies are detectable in human populations and are higher during the rainy season when mosquito populations are increased compared to dry seasons [26–32].

There are about 15 potentially antigenic proteins identified in the sialome of female of *Ae*. *aegypti* [33]. In particular, the putative 34 kDa family secreted salivary protein appears to be specific to *Aedes* genus validated by several studies: Antibody responses against N-term 34kDa were only detected in individuals who had been exposed to *Aedes* bites [24, 28, 34] and levels significantly declined shortly (~40 days) thereafter when the individuals were no longer exposed [28, 33]. N-term 34kDa IgG levels were also found to be higher during rainy season compared to dry season correlating with *Aedes* mosquito population densities [24, 34]. The levels of IgG against Nterm-34kDa did not differ significantly among DENV infected and non-infected individuals [24]. Nevertheless, N-term 34kDa IgG responses seems like a reliable measure of levels of exposure to *Aedes* mosquito bites, [35, 36] and may as well be applied to determine the heterogeneity of individual and population exposure [24, 34].

The evaluation of the serological biomarker, Nterm-34kDa, has, to the best of our knowledge never been attempted in Tanzania and more importantly, it has never been applied in regions where there is a risk of climate change-driven expansion of *Aedes* mosquitoes. In particular, highland areas of SSA require close monitoring as they are at an increased risk for expansion of arbovirus transmission following the increase in ambient temperature [22].

Lower Moshi, Tanzania, is a highland area with intense irrigation activities and evidence of circulating arboviruses mainly CHIKV [11]. Similarly, malaria transmission is low but persists throughout the year with seasonal fluctuations [37]. Both malaria and arboviruses are mosquito-borne diseases and their transmission is accelerated by more or less similar factors including meteorological factors such as ambient temperature, rainfall and humidity and non-climatic factors such as differences between human hosts, human migration and development projects [38, 39]. Nevertheless, the distribution and abundance of mosquitoes plays the central role in determining an increase or decrease in the transmission of malaria and arboviruses. Comparing the exposure to *Anopheles* and *Aedes* mosquitoes is important for understanding the mosquito to which individuals are highly exposed to and the areas with individual at high risk for both infections. This is important for vector control programs to direct where to intensify vector specific control interventions”.

We have previously evaluated the use of gSG6-P1 IgG as a serological biomarker for exposure to *Anopheles* mosquito bites in Lower Moshi and found significant variations in the seroprevalence between dry and rainy seasons, and significant variation in the seroprevalence by village of residence [26]. This gave us the opportunity, in the same setting to evaluate the human IgG response to the Nterm-34kDa antigen as a biomarker for exposure to *Aedes* bites and furthermore, explore the relationship between exposure to *Aedes* and *Anopheles* mosquitoes.

## Materials and methods

### Study site

Lower Moshi is located in the north eastern part of Tanzania within the Rural Moshi District at the foot of Mount Kilimanjaro (latitude 3°61’-3°68’S; longitude 37°32’-37°38’E). Lower Moshi is a highland area elevated to 800 meters above sea level. Three wards including Kahe, Arusha Chini and Mabogini make the Lower Moshi District. Large sugarcane plantations and a sugar factory are located within Lower Moshi. Other agricultural activities carried out in Lower Moshi include cultivation of rice, maize, beans, banana and animal keeping. Njoro and Rau rivers run across the villages and serve as sources of water for irrigation [40]. In Lower Moshi, malaria transmission occurs throughout the year with low parasitemia [37, 41] and prevalence below 0.1% [40]. The prevalence of dengue IgM and IgG among febrile ill individuals is 3.6% and 1.4%, respectively [11].

### Study design and sampling procedures

The initial study explored the levels of exposure to *Anopheles* mosquitoes’ bites. The design and sampling procedures have been described previously [26]. Briefly, three cross-sectional surveys were conducted in the study area in 2019. The baseline survey was conducted during the dry season (March) followed by two follow-up surveys during the end of the rainy season (June) and during a second dry season (September).

Three hundred and eight individuals aged from six months and above (7months-94years) were recruited from five selected villages in Lower Moshi. All 308 participants participated in the first dry season survey and of these, 201 participated in the rainy season and 204 participated in the second dry season survey.

### Ethical approval

Written informed consent was sought from the adult participants and parents or guardians consented on their children’s behalf. Approvals and permissions to carry out the surveys were obtained from Kilimanjaro Christian Medical University College (KCMUCo) Research Ethics and Review Committee (CRERC), certificate number 2409, District Executive Director (DED) of Moshi Municipal Council and from the local government leaders of the Lower Moshi study area.

### Data collection

Interviews were conducted during the first survey using electronic structured questionnaires in open data kit (ODK) application (ODK collect version 1.30.1; link: https://odk-collect.en.uptodown.com/android) to collect data on socio-demographic characteristics of the participants including age, sex and village of residence and education level. Whole blood samples (~500μL) were collected in ethylene-diamine tetra-acetic (EDTA) acid tubes (Becton Dickinson) during all three surveys. Rainfall data recorded in millimetres of rain from January 2019 to January 2020 was provided by the TPC Sugar Factory which is located within the villages selected for the study.

### Detection of IgG antibodies against N-term 34kDa

The whole blood samples were spin at 1500 x *g* for 5 minutes and obtained plasma was separated and stored at -80°C until use. As described in another study, through the use of blast family programs and genome databases, an N-terminal 19 amino acids peptide with amino acid sequence–HPIPAEDPAKQCNLSEDDL- belonging to a 34kDa protein family is both immunogenic and most specific to *Aedes aegypti* [34]. Synthetic form of the antigenic peptide N-term 34kDa (Catalogue number GPS_2958 Genepep, Saint Jean de Vedas-France), was dissolved in ultra-filtered water to a final working concentration of 20μg/mL.

Enzyme Linked Immuno-Sorbent Assay (ELISA) technique was performed as described elsewhere [42]. Briefly, ELISA plates (Sero-Well, Sterilin Appleton Woods Limited) were coated with N-term 34kDa antigen and incubated. Plates were blocked using protein free blocking buffer (Thermo Scientific, Meridian Road, Rockford, IL USA) then 20% plasma diluted in 1% PBS-Tween 20 was added in triplicates into two wells coated with antigen and one uncoated well followed by overnight incubation at 2–8⁰C. Biotinylated mouse anti-human antibody (Biosciences, San Diego, California, USA) was added and to detect bound human anti-N-term 34kDa IgG, horseradish peroxidase (HRP) streptavidin (Thermo Scientific) was added. 2,2’-Azinobis (3-ethylbenzothiazoline-6-sulfonic acid) (ABTS) (Sera Care, Gaithersburg, Maryland, USA) substrate was added and after sufficient colour development, optical densities (ODs) were read at 405 nm using Multi-Scan FC microplate photometer (Thermo Scientific) ELISA reader. The final ODs of each sample was obtained as ΔODs using the average OD of two antigen-coated wells subtracted the OD obtained from an uncoated well. Cut-offs for seropositivity were determined per plate as mean ΔODs plus two standard deviations of negative control plasma samples donated from seven unexposed Danish volunteers. These were included in each run, in which case cut-offs were determined per every run. For quality control, one positive control plasma sample obtained from *Aedes* bite exposed individuals were included in every run.

### Statistical analysis

Data were analysed using Stata Version 14 (StataCorp, Texas, USA) and GraphPad Prism version 9 (San Diego, California, USA) softwares. Chi square (χ^2^) was used to compare temporal variations in N-term 34kDa seroprevalence for all three surveys. Spearman correlation analysis was used to compare antibody levels against *Anopheles* and *Aedes* mosquitoes’ salivary antigens. The IgG levels against Nterm-34kDa were normalised to compare individual exposure levels. The association between N-term-34kDa seroprevalence and socio-demographic characteristics were determined using both univariate and multivariate logistic regression analyses. Multivariate logistic regression was performed with the inclusion of all variables with *p* < 0.2 in the univariate model. All differences were regarded statistically significant at *p* values < 0.05.

### Variables

The independent variables included the village of residence, age, sex, education level including individuals who had primary, secondary, tertiary education and those who never had formal education. The dependent variable was “N-term 34kDa seropositivity” defined as anti N-term 34kDa IgG levels above the negative value cut-offs.

## Results

### Characteristics of the study population

Description of study participants is as previously described [26]. In short, a total of 308 study participants were enrolled during the baseline survey conducted during the first dry season. Of them, 201 (65.3%) were followed in the rainy season and 204 (66.2%) in the second dry season majority of the participants being female (70%) and children aged between 6 and 15 years.

### Rainfall and Nterm-34 kDa seroprevalence

**Fig 1:**
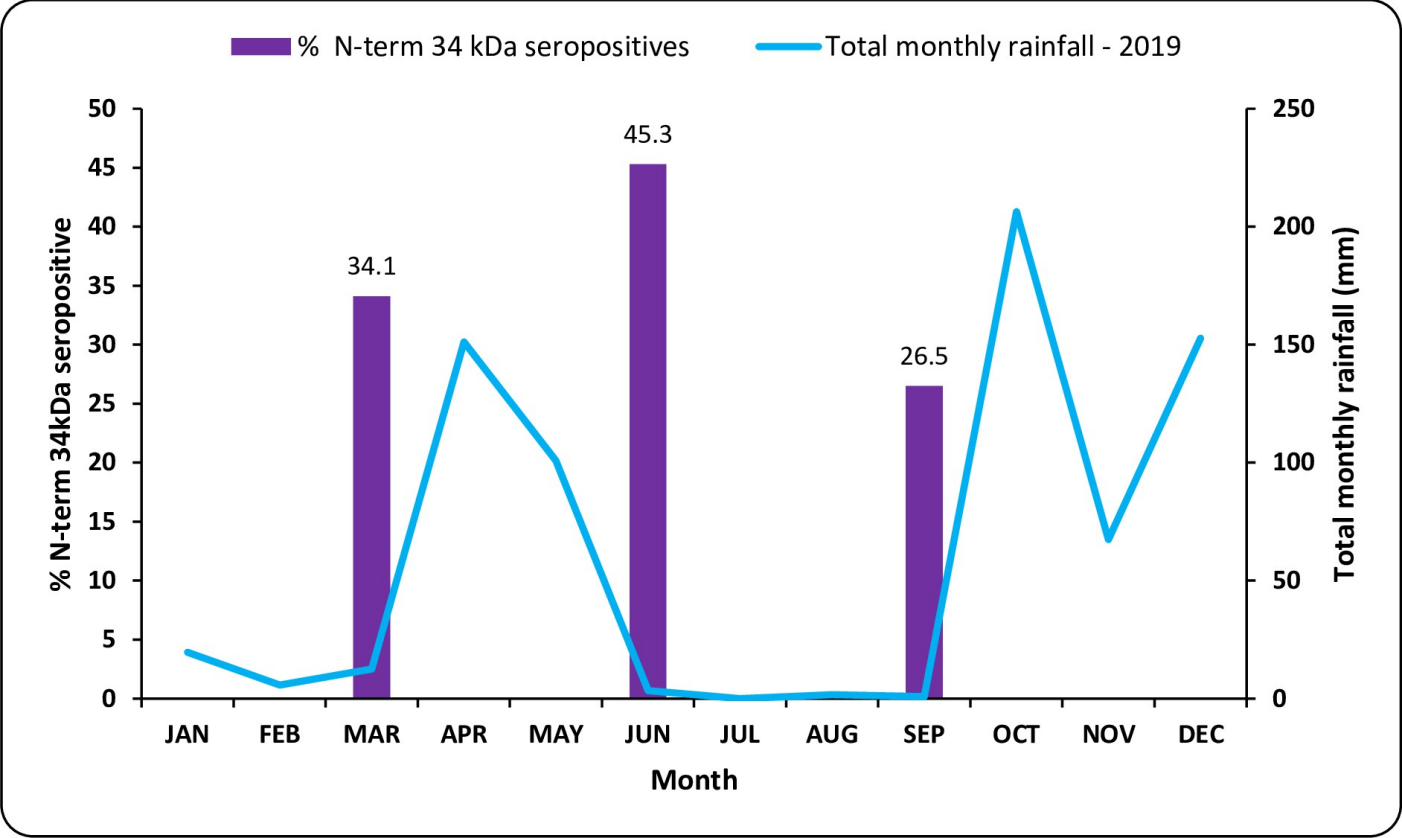
Percentage of Nterm-34 kDa seropositive and total monthly rainfall.

Total monthly rainfall for Lower Moshi varied over time. Rains were seen from March until June peaking in April, then followed a three-month period of drought from June to September, followed by yet another rainy season (Fig 1). Nterm-34 kDa seroprevalence also fluctuated over time. During the first dry season, Nterm-34kDa seroprevalence was 34.1% and significantly increased at the end of the rainy season to 45.3% (Chi square (χ^2^) = 6.42 *p* = 0.011). Between the rainy season and the second dry season, the seroprevalence significantly declined to 26.5% (χ^2^ = 15.12 *p*<0.001) (Fig 1 and S1 Fig).

### Spatial heterogeneity in Nterm-34 kDa seroprevalence

**Fig 2:**
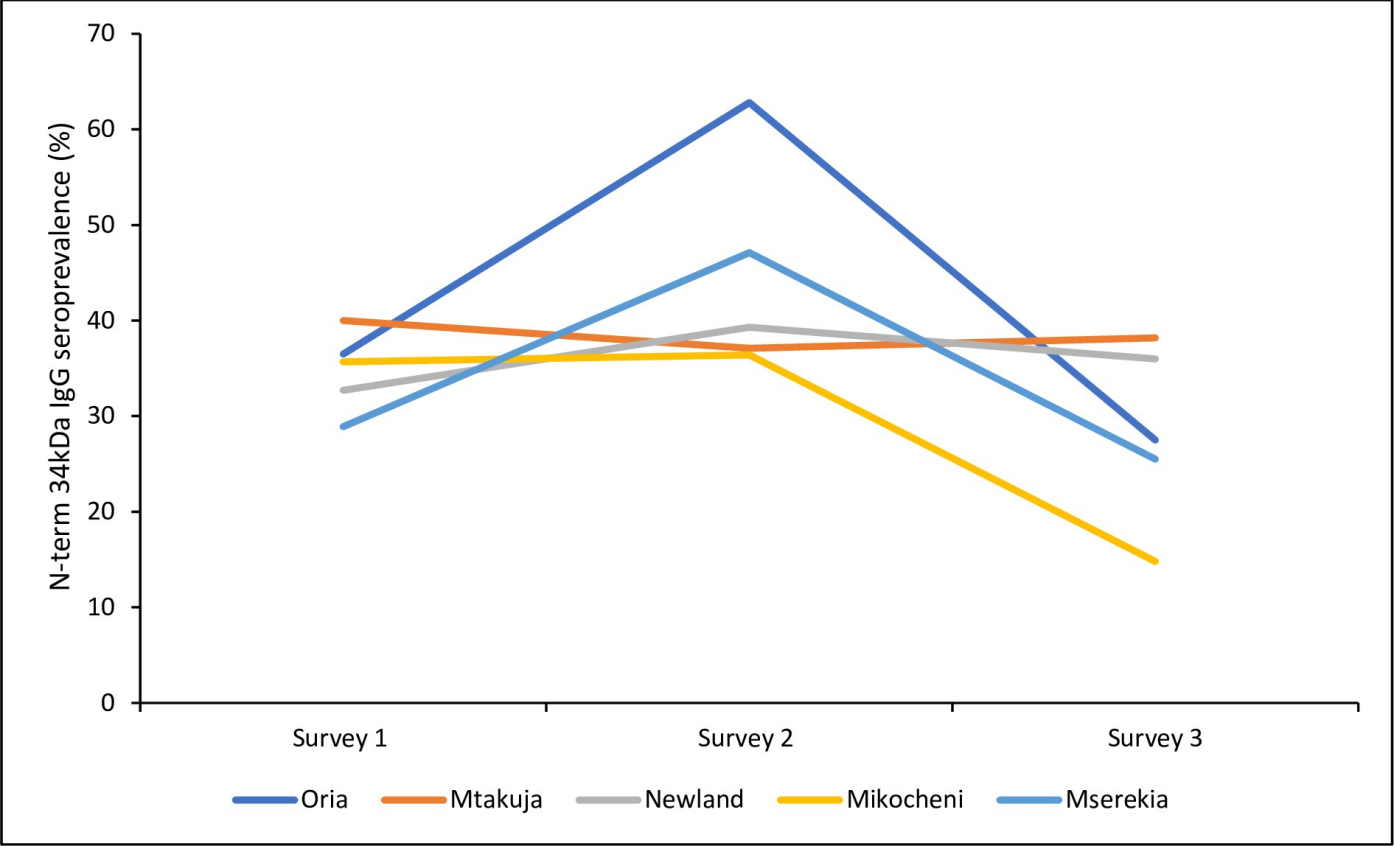
Nterm-34kDa seroprevalence by village during the three surveys.

Seroprevalence by village of residence was explored to compare heterogeneity in spatial levels of exposure (Fig 2). During the dry seasons (March (survey 1) and September (survey 3)) the seropositivity was highest among residents of Mtakuja village, while during the rainy season (June), seroprevalence was highest in Oria village. The seroprevalence varied significantly during the rainy season when comparing Oria against Mikocheni Mtakuja and Newland villages (χ^2^ test *p* = 0.014, 0.024 and 0.052 respectively) while there were no significant differences by village of residence during the dry season surveys were observed when comparing all villages during each season (χ^2^-test, *p*-values: first dry season 1, *p* = 0.755; second dry season 3, *p* = 0.116) were observed.

### Correlation between IgG levels against *Anopheles* and *Aedes* mosquitoes’ salivary antigens

**Fig 3:**
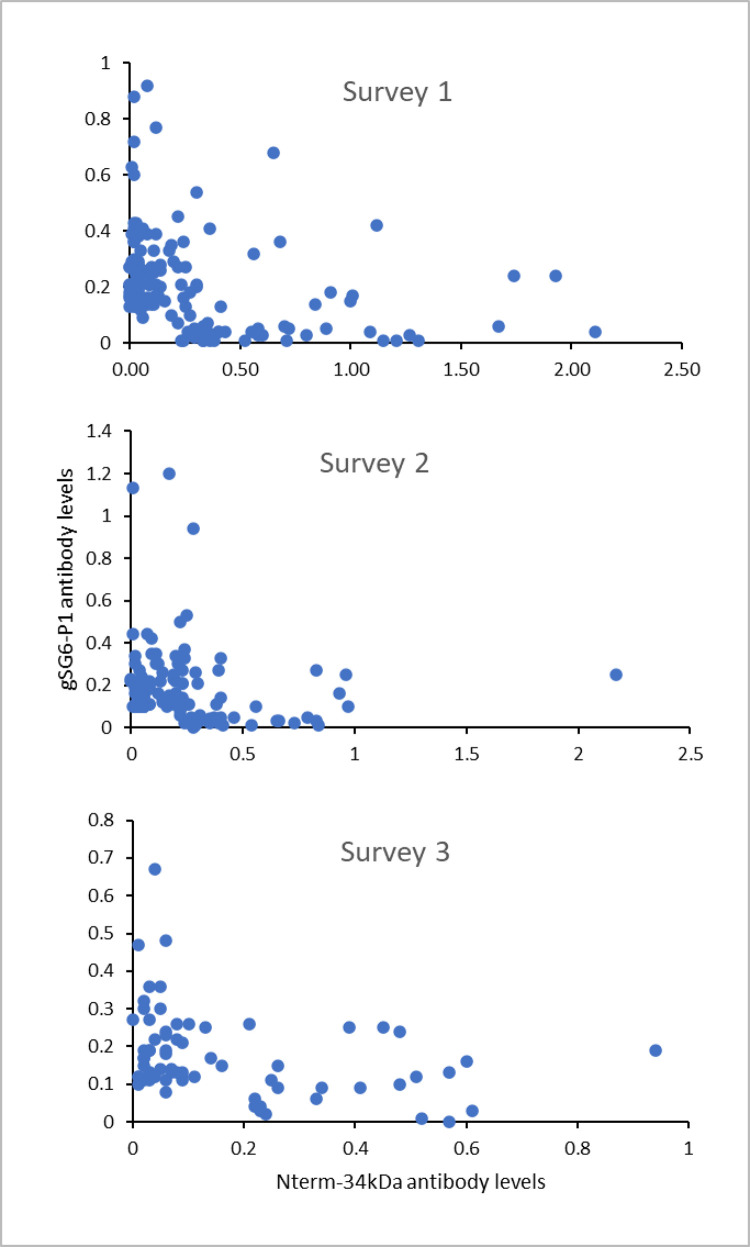
Comparison of exposure to *Anopheles* (antibody responses to gSG6-P1) and *Aedes* (antibody responses to Nterm-34) mosquito bites by survey. Participants that had a negative response to both gSG6-P1 and Nterm-34kDa antigens were excluded.

Using our previously published data on *Anopheles* salivary antigen [26], we explored the IgG levels against *Anopheles* and *Aedes* mosquitoes’ salivary antigens, gSG6-P1 and Nterm-34kDa. Plasma samples from the same individuals were used and, in the analysis, and participants who had negative response to both gSG6-P1 and Nterm-34kDa antigens were excluded. We only found a weak to moderate negative correlation (Fig 3) during surveys 1 (Spearman’s ρ = -0.4633, *p*<0.001), 2 (ρ = -0.3318, *p* = 0.0002) and 3 (ρ = 0.-3874, *p* = 0.0010) (Fig 3).

**Fig 4:**
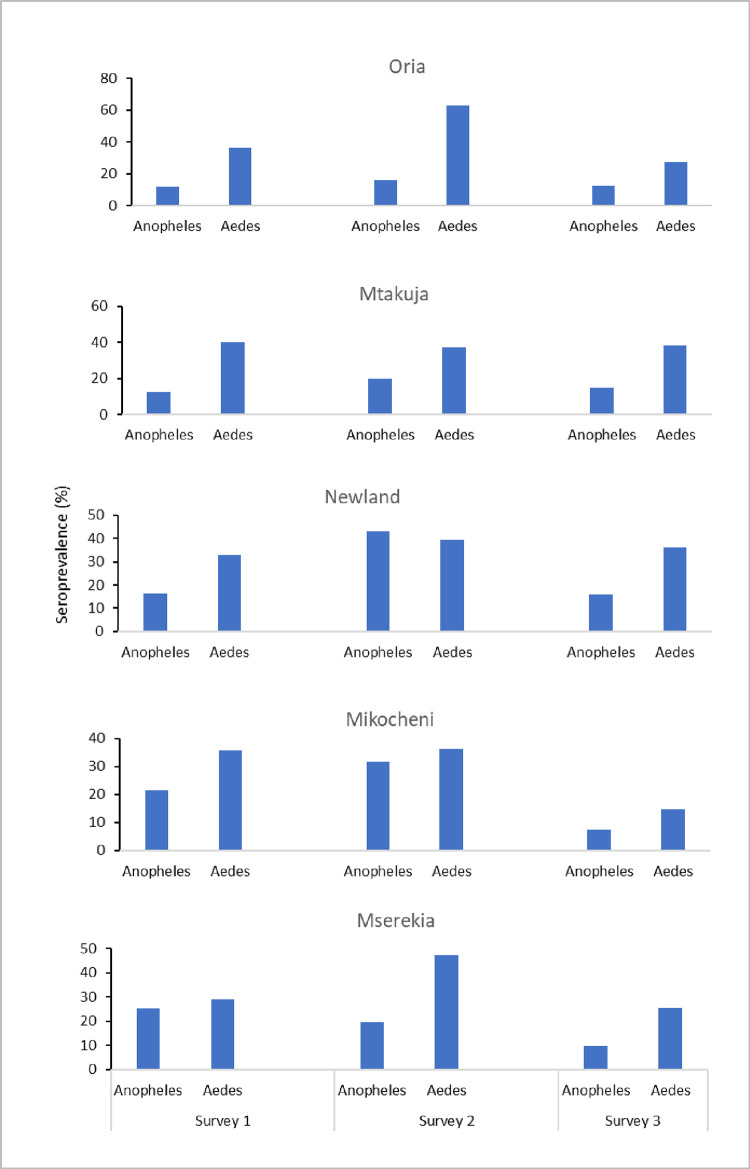
Comparison between gSG6-P1 IgG seroprevalence and Nterm-34kDa seroprevalence by village and survey.

We further compared the Nterm-34kDa and gSG6-P1 seroprevalence by village of residence (Fig 4). Except for the observation made in Newland village during the second survey, the seropositivity were generally higher with regards to response towards *Aedes* mosquitoes than *Anopheles* mosquitoes in all three surveys. Also, seropositivity for exposure to both *Anopheles* and *Aedes* mosquitoes were in most villages higher during the second survey than during the first and third surveys. Looking at exposure to *Anopheles* bites itself, the seropositivity was highest in Newland and Mikocheni and for *Aedes*, the villages with the highest seropositivity were contrarily Oria and Mserekia.

### Association between socio demographic characteristics of participants and exposure to *Aedes* mosquito bites

The associations between N-term 34kDa seropositivity and socio-demographic characteristics were explored by logistic regression (Table 1). During the first survey, both the crude and adjusted analyses showed that the risk of exposure to *Aedes* mosquito bites had no significant associations with village of residence, age, sex or level of education.

**Table 1:** Associations between socio-demographic characteristics of studied population and exposure to *Aedes* mosquito bites.

Variable	Survey 1				Survey 2				Survey 3			
	COR (95% CI)	*p*-value	AOR (95% CI)	*p*-value	COR (95% CI)	*p*-value	AOR (95% CI)	*p*-value	COR (95% CI)	*p*-value	AOR (95% CI)	*p*-value
**Village**												
Newland	1				1		1		1		1	
Oria	1.87 (0.5–2.7)	0.682			2.61 (1.0–6.9)	0.055	2.86 (1.0–7.8)	0.041	0.67 (0.2–2.0)	0.471	0.68 (0.2–2.0)	0.478
Mtakuja	1.38 (0.6–3.3)	0.473			0.91 (0.3–2.5)	0.862	0.91 (0.3–2.6)	0.856	1.10 (0.4–3.2)	0.861	1.11 (0.4–3.3)	0.846
Mikocheni	1.15 (0.5–2.4)	0.720			0.88 (0.3–2.3)	0.803	0.96 (0.4–2.6)	0.944	0.31 (0.1–0.9)	0.038	0.35 (0.1–1.1)	0.068
Mserekia	0.84 (0.3–0.8)	0.652			1.37 (0.5–3.5)	0.506	1.24 (0.5–3.3)	0.633	0.61 (0.2–1.7)	0.345	0.59 (0.2–1.7)	0.328
**Age**												
0–5	1		1		1		1		1		1	
6–15	1.25 (0.6–2.8)	0.578	0.51 (0.1–2.2)	0.373	0.40 (0.2–1.0)	0.049	0.40 (0.2–1.0)	0.057	0.44 (0.2–1.2)	0.095	0.46 (0.2–1.2)	0.126
16–30	1.89 (0.8–4.7)	0.172	2.18 (0.4–13.7)	0.404	0.30 (0.1–1.0)	0.058	0.25 (0.1–0.9)	0.036	0.87 (0.2–3.1)	0.829	0.79 (0.2–2.9)	0.723
31–45	1.69 (0.7–4.0)	0.224	1.92 (0.3–11.8)	0.480	0.64 (0.2–1.7)	0.380	0.59 (0.2–1.6)	0.315	0.42 (0.1–1.4)	0.155	0.40 (0.1–2.4)	0.150
46–65	1.38 (0.6–3.2)	0.443	1.65 (0.3–9.9)	0.581	0.89 (0.4–2.3)	0.812	0.85 (0.3–2.2)	0.731	0.99 (0.4–2.6)	0.984	0.88 (0.3–2.4)	0.798
66+	1.85 (0.7–4.7)	0.195	2.5 (0.4–15.1)	0.326	0.84 (0.3–2.3)	0.736	0.87 (0.3–2.6)	0.801	0.84 (0.3–2.6)	0.759	0.67 (0.2–2.3)	0.521
**Sex**												
Female	1				1				1			
Male	0.95 (0.6–1.6)	0.854			0.27 (0.7–2.3)	0.436			1.27 (0.7–2.5)	0.482		
**Education Level**												
No formal education	1		1		1				1			
Pupils at primary school	1.30 (0.6–2.7)	0.492	4.46 (0.8–25.3)	0.092	0.68 (0.3–1.6)	0.393			0.73 (0.3–2.0)	0.546		
Primary education and above	1.59 (0.8–3.1)	0.178	1.57 (0.8–3.3)	0.220	1.06 (0.5–2.3)	0.882			1.10 (0.4–2.8)	0.839		

COR- crude odds ratio, AOR- adjusted odds ratio, CI- confidence interval

During the second survey, in the crude model, the risk of exposure to *Aedes* bites was highest among residents of Oria village (COR = 2.61; 95% CI = 1.0–6.9; *p* = 0.055) and statistical significantly lower among individuals aged between six and 15 years (COR = 0.40; 95% CI = 0.2–1.0; *p* = 0.049) as well as 16–30 years (COR = 0.30; 95% CI = 0.1–1.0; *p* = 0.058) compared to the children aged between 0 and 5 years. In the adjusted analysis, the risk of exposure was statistical significantly higher among residents of Oria village (AOR = 2.86; 95% CI = 1.0–7.8; *p* = 0.041) and when adjusted for village of residence, risk of exposure was statistical significantly lower among individuals aged between 16 and 30 years (AOR = 0.25; 95% CI = 0.1 = 0.9; *p* = 0.036) compared to the other age groups.

During the third survey, in the crude analysis, risk of exposure to *Aedes* mosquitos’ bites was significantly lower among residents of Mikocheni village (COR = 0.31; 95% CI = 0.1–0.9; p = 0.038). Also, individuals aged between six and 15 years had the lowest risk of exposure (COR = 0.44; 95% CI = 0.2–1.2; p = 0.095) compared to the other age groups. In the adjusted analyses only living in Mikocheni village was borderline statistical significantly associated with reduced risk of exposure (AOR = 0.35; 95% CI = 0.1–1.1; p = 0.068).

## Discussion

This study evaluated the human antibody response to *Aedes* mosquito salivary antigen Nterm-34kDa in individuals living in a highland area of Lower Moshi, Tanzania, where there is evidence of circulating disease-causing arbovirus, particularly chikungunya [11]. The variation in IgG responses with rainfall patterns and villages of residence and the associations between IgG responses and socio-demographic characteristics of the studied population were determined. Responses to the *Aedes* salivary antigen were as well compared to previously published antibody responses to *Anopheles* salivary antigen gSG6-P1 [26].

Antibodies against Nterm-34kDa antigen were serologically detectable in the studied population and the responses varied with the seasons. Both individual and population IgG were significantly higher when measured just after the rainy season in June compared to the measurements done during the two dry seasons in March and September. Similar findings have been reported by other studies: e.g., studies conducted in West Africa have shown that Nterm-34kDa seroprevalence varied with season and increased during rainy seasons [34, 42–44].

The current study found that seroprevalence by village increased during the rainy season for most villages. However, while all villages had a clear tendency of increase during the rainy season and decrease during dry season, Mtakuja had persistently high seroprevalence throughout the year. This is probably due to the location of the village; adjacent to the intense sugarcane irrigation scheme which may increase *Aedes* mosquitoes breeding sites as there may be more stagnant water collections in cavities capable of retaining water suitable for larvae development. Sugarcane irrigation areas are also likely to be more wet and humid during the dry season which is one of the main climatic factors contributing to *Aedes* abundance and their associated disease transmission [45]. Unfortunately, *Aedes* mosquitoes were not collected in this current study, and could not be compared to the antigen levels measured. A study conducted in a palm and rubber agriculture setting in south-eastern Cote d’Ivoire reported similar findings where higher IgG responses were seen to be high and persistent with no apparent seasonality in rubber and palm intense cultivation villages compared to a village with less intense agricultural practices [42].

The individual IgG responses against *Anopheles* and *Aedes* salivary antigens were compared and there was only a weak to moderate negative correlation between individual responses against gSG6-P1 and Nterm-34kDa were found. This indicated that, in Lower Moshi, individuals exposed to *Anopheles* mosquitoes’ bites were not necessarily exposed to *Aedes* mosquitoes’ bites. Moreover, this shows that individual exposure to *Anopheles* bites is independent of exposure to *Aedes* bites and this is most likely due to the important differences in the behaviour of the mosquitoes, such as breeding, feeding and resting habits. *Anopheles arabiensis* is the dominant species in lower Moshi and feeds both indoor and outdoor, and bites mostly at night [46] and breeds in water pools from rains or irrigation canals, ditches and natural containers [47]. On the other hand, *Aedes* mosquitoes rest and bite outdoors during morning and evening hours [48] and breed in either domestic or artificial water holding containers [42, 49].

In this study, it was observed that seroprevalence for *Aedes* salivary antigen were higher compared to seroprevalence for *Anopheles* mosquitoes. This is possibly due to variation in biting habits and host preference of the two different mosquitoes where *Aedes* bites preferably during the day [48] when people are less protected from vector bites and due to that *Aedes* are almost exclusively anthropophilic [50] while *An*. *arabiensis* are mostly zoophilic and mostly bites at night when most individuals are tucked under bed nets [51].

The seroprevalence by village of residence were also compared regarding exposure to both mosquitoes. The findings show that both gSG6-P1 and Nterm-34kDa seroprevalence were higher during the rainy seasons compared to the dry seasons for most villages. However, during the rainy season, exposure to *Anopheles* mosquitoes were highest in Newland village and lowest in Oria village, while exposures to *Aedes* mosquito bites were highest in Oria village compared to the rest of the villages. Our previous study observed that *Anopheles* mosquitoes were found to be highest in number in Oria village compared to the rest of the villages although the level of exposure to *Anopheles* bites was the lowest in Oria village compared to the rest of the villages [26]. One possible explanation of this apparent paradox is that Oria village residents had the highest number of individuals owning and using bed nets compared to the residents from the other villages [26], however this does not protect the residents from outdoor biting *Aedes*. Overall, this suggests that Oria village probably produces the largest numbers of both *Anopheles* and *Aedes* mosquitoes.

The current study explored the associations between socio-demographic characteristics (including village of residence, age, sex, and education level) and Nterm-34kDa IgG levels In dry seasons, there were no significant associations found between Nterm-34kDa seropositivity and various characteristics explored, probably because the dry seasons surveys had few mosquitoes breeding sites and thus, low mosquito populations, however, unfortunately the abundance of *Aedes* mosquitoes was not explored further.

During the second survey conducted just after the long rains, seroprevalence was significantly higher in Oria village compared to the rest of the villages. We observed that, Oria village has more vegetation cover compared to the rest of the villages, which possibly favours the presence of *Aedes* mosquitoes during the rainy seasons by making the environment more cool and humid as preferred by *Aedes* mosquitoes [45]. Furthermore, a study conducted in an urban setting in Zanzibar found that the presence of vegetation was significantly associated with the presence of immature stages of *Aedes aegypti* [52]. Another study conducted in both rural and urban Kenyan setting also reported the strongest risk factor for *Aedes* pupa abundance were the presence of bushes, tall around the houses [53]. Thus, the combined effect of rains and more dense vegetation cover may be the probable reason for the significant increase in *Aedes* mosquitoes’ abundance and thus exposure to *Aedes* mosquito bites in Oria village, in particular.

Also during the rainy season, the risk for exposure to *Aedes* mosquito bites was significantly low among individuals aged between 16 and 30 years compared to those aged between 0 to 5 years. This association cannot be ascertained due to the complexity of age related behaviour which may influence exposure to mosquito bites. Contrary to our findings, a study conducted in Thailand found that the risk for exposure to *Aedes* mosquito bites to be significantly higher among old aged individuals (60–69 years) [54].

## Conclusion and recommendation

This study’s findings support that antibody responses towards the *Aedes* mosquito salivary peptide Nterm-34kDa vary with season and geographic location. More individuals are exposed to *Aedes* mosquitoes than *Anopheles* mosquitoes and those exposed to *Aedes* mosquito bites are not equally exposed to *Anopheles* mosquito bites. Further studies which can compare the abundance of *Aedes* mosquito to the level of exposure are recommended to provide further insights into the value and potency of this tool. Standardization and optimization of methods for the detection and quantification of these antibodies are needed before this tool can be adopted for use by vector control programmes.

## Supporting information

S1 Fig Normalised individual N-term 34kDa IgG levels measured at three different time points representing different seasons. The bars show the median values.
(TIF)

S1 Dataset Nterm-34kDa IgG antibody response in Lower Moshi. (XLS)

S1 File Questionnaire English version. (DOCX)

S2 File Questionnaire Swahili version. (DOCX)
