# Supplementary figures and images for: Human IgG responses to Aedes mosquito salivary peptide Nterm-34kDa and its comparison to Anopheles salivary antigen (gSG6-P1) IgG responses measured among individuals living in Lower Moshi, Tanzania
Source: PLoS One. 2022 Oct 27;17(10):e0276437. doi: 10.1371/journal.pone.0276437 (PMC9612500; doi:10.1371/journal.pone.0276437)

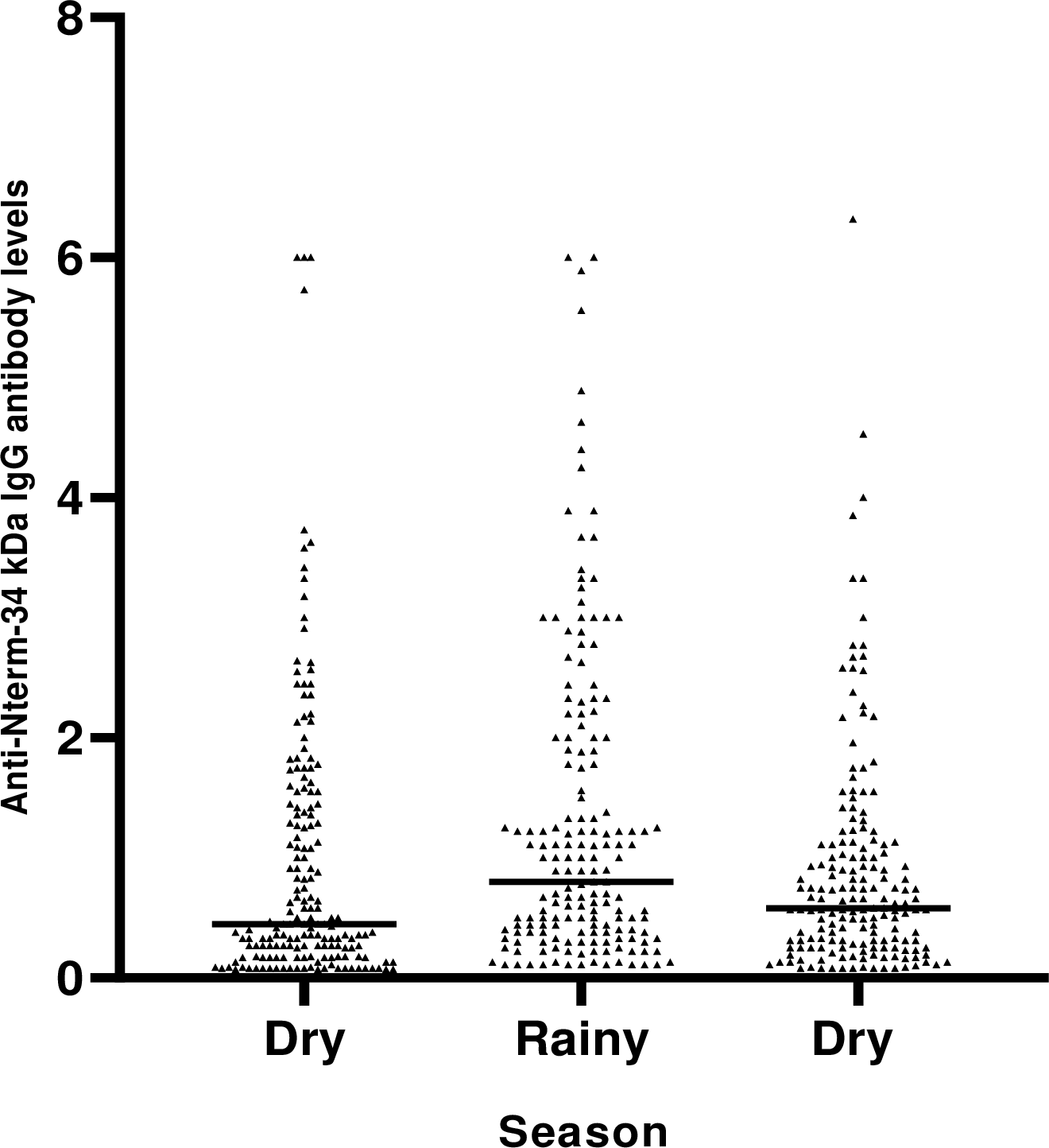

Supplement: S1 Fig — The bars show the median values. (TIF) [file pone.0276437.s001.tif]
